# Unexpected thymoma in a challenging case of hyperparathyroidism

**DOI:** 10.1002/ccr3.2842

**Published:** 2020-06-18

**Authors:** Federico Raveglia, Loredana De Pasquale, Ugo Cioffi, Giorgio Ghilardi, Matilde De Simone, Monica Falleni, Alessandro Baisi

**Affiliations:** ^1^ Thoracic Surgery ASST Santi Paolo e Carlo University of Milan Milan Italy; ^2^ Endocrine Surgery ASST Santi Paolo e Carlo University of Milan Milan Italy; ^3^ Pathology ASST Santi Paolo e Carlo University of Milan Milan Italy

**Keywords:** hyperparathyroidism, parathyroid, thoracic surgery, thymoma

## Abstract

We report the case of a woman with primary hyperparathyroidism suspected of mediastinal ectopic parathyroid adenoma revealed to be a thymoma. Our aim was to focus on some possible criticisms in distinguishing between ectopic parathyroid and thymus.

## Graphical abstract

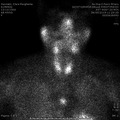

## 1 INTRODUCTION

Very few cases of concomitant thymoma and parathyroid adenoma have been reported, showing that this pattern is rare and challenging. We report a case of primary hyperparathyroidism in a woman with mediastinal tumor. False‐positive imaging, intraoperative findings, and changing surgical strategy make the case very educational.

## 2 CASE REPORT

**Figure 1:**
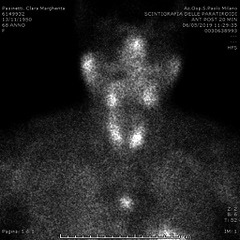
Tc 99 m sestamibi scan showing a main pathologic increased uptake on immediate imaging in the anterior mediastinum

**Figure 2:**
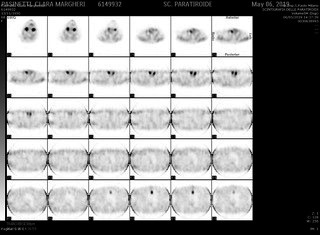
Tc 99 m sestamibi scan showing a main pathologic increased uptake on immediate imaging in the anterior mediastinum

A 68‐year‐old woman under medical supervision from 2013 for osteoporosis presented a clinical history characterized by symptomatic kidney stones, bone fractures, diarrhea, abdominal pain, dysphagia, and dyspnea. In 2019, she performed the first check at Endocrine Surgery Division because of gradual osteoporosis worsening and recurrent urologic symptoms. Laboratory results [serum calcium level of 8.99 mg/dL (8.5‐10‐5 mg/dL), ionized calcium of 4.69 mg/dL (4.64‐5.28 mg/dL), PTH of 191.50 pg/mL (10‐65 pg/dL), and vitamin D of 40 ng/mL (20‐50 ng/mL)] confirmed diagnosis of primary hyperparathyroidism with normal calcium serum level. Tc 99 m sestamibi showed a main pathologic increased uptake on immediate imaging and a decreased signal on delayed imaging in the anterior mediastinum and a slight uptake at the lower third of left thyroid lobe (Figures 1, 2). This second uptake was also present at Tc‐99 m pertechnetate scan as in case of hyperfunctional thyroid. CT scan showed a tumor mass of 18 mm in the upper anterior mediastinum, behind the sternum. The most likely clinical diagnosis was primary hyperparathyroidism and ectopic mediastinal adenoma. Based on disease progression, she was referred to surgery. Preoperative evaluation was completed with neck ultrasound that found a couple of thyroid nodules at the right lobe.

She was referred to our Thoracic Surgery Division for removal of the mediastinal tumor. The operative strategy, shared with the patient, was to approach the tumor by thoracoscopy and remove it. Only in case of failure to decrease the intraoperative value of the PTH, would a cervicotomy have been performed for the removal of the parathyroid glands. Just before surgery, PTH was 190.1 pg/mL. Patient was positioned in supine decubitus, and general anesthesia was done by double‐lumen intubation. We performed a right uniportal video‐assisted thoracic surgery approach through a 4‐cm‐long anterolateral thoracotomy at 4th intercostal space. Once detected in the anterior upper mediastinum, the tumor was radically resected by the use of harmonic scalpel. Frozen section showed a mass with a nodular pattern of growth, composed mainly by an epithelial component mixed with a patchy lymphoid component. Epithelial cells were predominantly round to oval, rarely spindle cell elements, with round‐ or oval‐shaped nuclei and inconspicuous nucleoli, arranged in solid sheets, nests, and occasional pseudoglandular structures. The lymphocytic component was mainly localized at the periphery of the mass. Occasional cystic spaces could be observed. Necrosis and mitotic figures were absent. These morphological aspects during intraoperative examination were considered consistent with the clinical suspicion of an ectopic pathological parathyroid gland. Intraoperative PTH assay was used to indirectly confirm diagnosis. Laboratory values were obtained after a 10‐minute interval from tumor removal. Surprising, PTH drop was slight (PTH = 149.3 pg/mL). It was repeated at 30 minutes, but value increased (259.5 pg/mL).

Therefore, endocrine surgeons performed neck exploration through a transverse cervical incision. Left upper parathyroid gland appeared enlarged and was removed. Frozen section was suggestive of hypercellular parathyroid. Also, the left lower gland appeared enlarged and was removed; pathology was suggestive of hypercellular parathyroid and residual thyme tissue. Since parathyroid hyperplasia typically involves all four glands and the right lower gland was increased in size, it was removed as well. Frozen section was suggestive of hypercellular parathyroid. Final PTH was 35 pg/mL. Postoperative was uneventful. Oral calcium was administered, and she was discharged in the 4th postoperative day.

Contradicting intraoperative findings, at definitive examination the mass revealed no immunostaining for PTH but immunoreactivity was coherent with type AB thymoma (sec WHO). Definitive diagnosis was primary hyperparathyroidism in parathyroid hyperplasia and concurrent thymoma. Masaoka stage I did not necessitate further intervention.

## 3 DISCUSSION

Parathyroid adenoma and thymoma are uncommon diseases. However, the simultaneous presentation of both tumors has been described, sometimes with ectopic cervical thymus or mediastinal parathyroid^1^, ^2^, ^3^, ^4^, ^5^ pattern. This relationship could be explained based on common embryologic developmental since lower parathyroid glands and thymus originate from the third pharyngeal pouch; therefore, the parathyroid glands separated from thymus move posteriorly to the thyroid.

Among concurrent cases reported in literature, the most of patients presented hyperparathyroidism and coexisting or subsequent myasthenia gravis, suggesting for a mediastinal disease. By contrast, in few cases, thymoma was accidentally found during cervicotomy, thanks to its ectopic position. Therefore, Verroiotou et al,^6^ describing the first Greek case, preoperatively seek for thymic lesion in patients with primary hyperthyroidism since silent thymoma could coexist.

However, the diagnosis can be even more challenging. In 2014, Cunningham et al^7^ published a case report very similar to ours; a woman affected by primary hyperparathyroidism underwent Tc 99m sestamibi and CT scan demonstrating a mass in the anterior mediastinum consistent with ectopic parathyroid adenoma and a slight asymmetric uptake in the right inferior thyroid gland consistent with multinodular goiter. Transternal resection of the mass was performed with immediate drop in PTH levels. Final histology showed unexpected AB thymoma Masaoka pT2a. However, one month later PTH levels increased again because of an inferior right cervical parathyroid adenoma, detectable at this point at scintigraphy.

As in their clinical case, we had to deal with a false‐positive for original mediastinal parathyroid adenoma Tc 99 m sestamibi scan and false‐negative for cervical adenoma/hyperplasia. In both cases, a mediastinal mass suspected of ectopic parathyroid was found to be a thymoma. Scintigraphy sensitivity and positive predictive values are 82.1% and 93%, respectively. Usually, false uptake has been documented in both benign and malignant disease with elevated mitochondria such as in thyroid tissue but also in other tissues, such as lung, brain, bone, thymus, or lymphatic. Thymomas and other mediastinal tumors with pathological technetium Tc 99m sestamibi uptake have been already described and investigated, so much so that Fiorella et al^8^ proposed to correlate uptake levels with WHO classification and Masaoka stage.

Our case requires the utmost care in reading the scintigraphy in those clinical contexts in which patients present hyperparathyroidism and mediastinal tumor. Indeed, an unexpected thymoma or a silent cervical parathyroid adenoma could not be excluded in advance. Therefore, in these rare cases, the preoperative assessment should always be completed with the chest CT scan and the ultrasound of the neck.

Moreover, we underline the role of intraoperative PTH levels measurement and frozen section in influencing surgical strategy. The pathologist should always be accurately informed in advance about patients’ clinical history and about specific need of a differential diagnosis between thymus or parathyroid tissue. Indeed, surgical strategy could be modified in midcourse, based on mediastinal sample examination findings and PTH levels. In the case of thymoma, complete removal of mediastinal adipose tissue should be considered, whereas in the case of persistent PTH high levels, involvement of endocrine surgeons and neck exploration are mandatory.

## CONFLICT OF INTEREST

None declared.

## AUTHOR CONTRIBUTIONS

All authors: were involved in substantial contributions to conception and design, acquisition of data, or analysis and interpretation of data; drafted the article or revised it critically for important intellectual content; and involved in final approval of the version to be published.

## CONSENT FOR PUBLICATION

Written informed consent was obtained from the patient for publication of this case report and any accompanying images. A copy of the written consent is available on request.

## Data Availability

Data sharing not applicable to this article as no datasets were generated or analyzed during the current study.
